# Intimate partner violence and its association with alexithymia in pregnant women from two hospitals in Cusco, Peru: an analytical cross-sectional study

**DOI:** 10.17843/rpmesp.2026.432.16064

**Published:** 2026-06-30

**Authors:** Ana Claudia Santander-Cahuantico, Maycol Suker Ccorahua-Rios, Jorge Luis Cabezas Límaco, Victor Hugo Arias Chavez, Alejandro Joaquin Santander-Cahuantico, Franklin Miranda-Solis

**Affiliations:** 1 Escuela Profesional de Medicina Humana, Universidad Nacional de San Antonio Abad del Cusco, Cusco, Perú.; 2 Unidad de Cuidados Intermedios Adultos, Departamento de Emergencia y Cuidados Críticos, Hospital Antonio Lorena del Cusco, Cusco, Perú.

**Keywords:** Intimate Partner Violence, Alexithymia, Mental Health, Pregnant Women

## Abstract

**Objetives.:**

To determine the association between intimate partner violence and alexithymia in pregnant women treated at two hospitals in Cusco, 2025.

**Materials and methods.:**

A cross-sectional analytical study was conducted, which included 286 pregnant women treated in the outpatient clinics of Gynecology and Obstetrics at Hospital Antonio Lorena and Hospital Regional del Cusco between February and April 2025. Direct interviews were applied using the Toronto Alexithymia Scale (TAS-20) and the Woman Abuse Screening Tool (WAST). Statistical analysis was performed using the generalized linear model (GLM) with Poisson distribution, logarithmic link function, and robust variance, calculating adjusted prevalence ratios (aPR).

**Results.:**

286 pregnant women were included. 71.7% presented alexithymia and 55.6% reported intimate partner violence (IPV). The study reveals that the prevalence of alexithymia was 50% higher in pregnant women with IPV (aPR: 1.50; 95% CI: 1.26-1.79; p<0.001), adjusted for origin, educational level, employment status, economic situation, number of gestations, and pregnancy planning.

**Conclusions.:**

Intimate partner violence was significantly associated with a higher prevalence of alexithymia in pregnant women treated at two hospitals in Cusco. Pregnant women exposed to violence showed a 50% higher prevalence of alexithymia, even after adjusting for sociodemographic and obstetric variables.

## INTRODUCTION

Intimate partner violence (IPV) constitutes a serious public health problem due to its high prevalence and the multiple negative consequences it causes on the physical and mental health of victims ^1^. According to the Pan American Health Organization (PAHO), approximately 25% of women aged 15 to 49 have experienced physical and/or sexual IPV at least once in their lives ^2^. This burden is especially high in low and middle-income countries, where the highest prevalence of violence against women is concentrated ^3^. In Peru, data from the 2024 Demographic and Family Health Survey (ENDES) reported that 52.0% of women had experienced some type of IPV at least once ^4^, which highlights its importance in the national context.

This problem takes on an even more critical dimension during pregnancy, since pregnant women constitute a particularly vulnerable population ^5^. Their exposure to violence has been linked to obstetric complications, such as fetal trauma, preterm birth, spontaneous abortion, low birth weight, and increased risk of maternal mortality; as well as with mental health problems, including an increased risk of depression, anxiety, post-traumatic stress, and substance abuse ^5^^-^^7^.

In this context, alexithymia, understood as the difficulty in identifying, describing, and expressing emotions ^8^, emerges as a crucial factor. It has been described as a possible coping mechanism in situations of chronic stress, such as violence, and could mediate or exacerbate emotional effects during pregnancy ^9^. However, although there is evidence of a significant relationship between IPV and alexithymia in adult women, the literature has not yet explored this association in pregnant women ^10^^-^^12^.

This research gap is particularly relevant in Peru, where, despite institutional efforts to eradicate IPV, the levels remain concerning ^(^^4^. The situation is more critical in regions like Cusco, where levels exceed the national average and economic, social, and cultural factors can amplify the effects of violence, as well as limit access to adequate mental health services ^13^. The scarcity of studies on the interrelationship between IPV and alexithymia during gestation in this context leaves a critical knowledge gap. This constitutes not only a constant challenge for the national health system, but also a global concern aligned with the Sustainable Development Goals, which promote gender equality and the eradication of all forms of violence against women ^14^. Understanding this relationship is urgent, given the evidence suggesting a link between IPV and elevated levels of alexithymia ^(15-20)^ and its negative implications for mental health and the quality of the maternal-fetal bond ^21^.

Therefore, this study aimed to determine the association between intimate partner violence and alexithymia in pregnant women attended in two hospitals in Cusco.

**KEY MESSAGES**

Motivation for the study. The high rates of intimate partner violence during pregnancy and the scarce evidence on its impact on the ability to process emotions (alexithymia) motivated the conducting of this research.

Main findings. The study found a clear association between suffering violence and presenting alexithymia in pregnancy, directly linking mistreatment with emotional difficulties.

Public health implications. These findings highlight the need to strengthen compliance with national strategies for the detection and comprehensive care of violence against women during prenatal care.

## MATERIALS AND METHODS

### Design and study population

An observational, cross-sectional analytical study was conducted, prepared according to the guidelines of Strengthening the Reporting of Observational Studies in Epidemiology (STROBE^) (^^22^, in pregnant women treated in the outpatient clinics of Gynecology and Obstetrics of two level III public hospitals in Cusco belonging to the Ministry of Health (MINSA). Both hospitals were selected for being regional referral centers that concentrate a high demand for care with comparable healthcare characteristics. Data collection was carried out between February and April 2025.

The population consisted of pregnant women over 18 years of age who provided their informed consent. Those with cognitive impairment, severe obstetric complications, hospitalization, or emergency care were excluded. The sample size was calculated using the Epi Info™ version 7.2.5.0 software for a cross-sectional analytical study of proportion comparison. Due to the absence of previous local studies that evaluated the association between intimate partner violence and alexithymia in pregnant women, the parameters reported by Chandan *et al*. ^23^ were used as reference, considering a 95% confidence level, an 80% statistical power, a non-exposed/exposed ratio of 4:1, an expected outcome proportion of 23.9% in non-exposed and 45.1% in exposed. A minimum size of 260 participants was obtained. Additionally, a 10% increase was made for possible losses, finally obtaining a sample of 286 pregnant women. Participants were included by consecutive non-probabilistic sampling.

### Data collection

Data collection was performed between February and April 2025 in the outpatient clinics of Gynecology and Obstetrics of the participating hospitals. Pregnant women were invited to participate at the end of their prenatal visit. Before their inclusion in the study, compliance with eligibility criteria was verified, and written informed consent was obtained.

During the study period, 297 pregnant women who met the eligibility criteria were invited to participate. Eight pregnant women declined to participate after receiving information about the study. Of the 289 who accepted to participate and completed the informed consent process, three were excluded from the analysis due to incomplete information in the questionnaires. Finally, 286 pregnant women were included.

The instruments were applied by surveyors previously trained in the study objectives, the standardized administration of the questionnaires, and the handling of sensitive information related to intimate partner violence. Interviews were conducted individually in an environment independent of the consultation area, ensuring privacy and confidentiality throughout the process.

The average application time was approximately 15 minutes per participant. At the end of each interview, the questionnaires were reviewed by the interviewers to verify the integrity of the collected information.

### Instruments and variables

Data collection was performed through a survey distributed in the following sections: sociodemographic and gynecological-obstetrical characteristics, the Toronto Alexithymia Scale (TAS-20), and the Woman Abuse Screening Tool (WAST).

Sociodemographic variables included the age of the pregnant woman, place of origin, marital status, educational level, employment status, and economic status. Origin was classified as urban or rural according to the usual place of residence reported by the pregnant woman. Economic status was evaluated by self-reporting the approximate monthly household income and categorized into low (1300-2400 soles), medium (2401-6999 soles), and high (≥ 7000 soles) levels. Gestational trimester was recorded according to gestational age estimated by last menstrual period or obstetric ultrasound. The number of gestations was determined by participant self-report and included the current pregnancy. Pregnancy planning by self-report corresponded to the assessment of whether the current pregnancy was desired and planned before conception.

The outcome variable was Alexithymia, evaluated using the TAS-20 Spanish version validated by Perez-Rincon *et al*. ^24^; composed of twenty items, and presents a Cronbach's alpha of 0.875, which indicates high internal consistency. The total score ranges from 0 to 100, classified as: absence of alexithymia (≤51 points), borderline alexithymia (52‒60 points), and alexithymia (≥61 points) ^24^. For statistical analysis, the results were dichotomized into two categories: absence of alexithymia (≤51 points) and presence of alexithymia (≥52 points).

For the evaluation of IPV, the short version of the WAST (Woman Abuse Screening Tool) ^25^ was applied, adapted to the Peruvian context and incorporated by MINSA in the Technical Guide for Mental Health Care for Women in Situations of Violence (2021) ^26^. This version consists of eight Likert-type items, with 100% sensitivity, 96.4% specificity, and a Cronbach's alpha of 0.91 ^25^. The total score ranges between 8 and 24 points. A score of ≥15 was considered a positive screening for intimate partner violence. Additionally, following the instrument's interpretation criteria, participants who answered affirmatively to questions related to physical violence («Do arguments end in blows, kicks, or shoves? ») or verbal violence («Does your partner insult, yell at, humiliate, or verbally demean you? ») were also classified as positive, even when the total score was less than 15 ^26^.

### Statistical analysis

The data obtained were processed in Microsoft Excel® 365 and analyzed using Stata v15.1 software (StataCorp, College Station, TX, USA). A quality control was performed to verify the absence of missing data, duplicates, empty cells, or inconsistencies in the database.

In the univariate analysis, absolute and relative frequencies of the study variables were calculated. Subsequently, in the bivariate analysis, the association between intimate partner violence and alexithymia, as well as with sociodemographic and gyneco-obstetric variables, was evaluated. For this, a generalized linear model (GLM) with Poisson distribution, logarithmic link function, and robust variance was used to estimate prevalence ratios (PR) and their respective 95% confidence intervals. Subsequently, multivariate analysis was performed using the same model to obtain adjusted PR between intimate partner violence and alexithymia. The variables included in the adjusted model were: origin, educational level, employment status, economic situation, number of gestations, and pregnancy planning, which showed a statistically significant association in the bivariate analysis (p<0.05). Likewise, multicollinearity was assessed using the variance inflation factor (VIF), with values <6 considered acceptable. A p-value < 0.05 was considered significant.

### Ethical aspects

The study had institutional authorization from Hospital Regional del Cusco and Hospital Antonio Lorena, was evaluated and approved by the Institutional Research Ethics Committee and the Office of Training, Teaching, and Research (code: 1107-HRC-OCDI). Likewise, informed consent was obtained from all participants, guaranteeing the anonymity and confidentiality of the information. Pregnant women who presented with IPV and alexithymia were provided guidance on the available institutional services and psychological follow-up was recommended by the Psychology service of the participating establishments.

## RESULTS

During the recruitment period, 297 pregnant women eligible to participate in the study were evaluated. Eight refused to participate, and three were excluded due to incomplete information in the questionnaires. Finally, 286 pregnant women attended in the outpatient services of Gynecology and Obstetrics of the Antonio Lorena Hospital (58.4%) and the Regional Hospital of Cusco (41.6%) were included. Of the total participants, 71.7% (n = 205) presented alexithymia, while 28.3% (n=81) did not.

### Sociodemographic characteristics

85% of the participants belonged to the 18 to 34 years age group, a similar proportion among pregnant women with and without alexithymia. Urban origin predominated (59.8%), with no marked differences between both groups. Regarding civil status, the majority identified as cohabiting (90.2%).

Regarding educational level, 58% of pregnant women had completed secondary education, while 35.3% had higher education. It was observed that 65.4% of the group of pregnant women with alexithymia had a secondary level, whereas among pregnant women without alexithymia, higher education predominated (56.8%). In relation to employment status, the majority were unemployed (68.9%) and belonged to a low economic stratum (66.8%) (table 1).

**Table 1:** Distribution of sociodemographic, gyneco-obstetric, and intimate partner violence characteristics in pregnant women, according to the presence of alexithymia (N=286).

Characteristics		Total sample N=286	Pregnant Woman with Alexithymia n=205	Pregnant woman without alexithymia n=81
		n (%)	n (%)	n (%)
Hospitals				
	H. Regional del Cusco	119 (41.6)	83 (40.5)	36 (44.4)
	H. Antonio Lorena	167 (58.4)	122 (59.5)	45 (55.6)
Age				
	18-34 years	243 (85.0)	171 (83.4)	72 (88.9)
	35 or more	43 (15.0)	34 (16.6)	9 (11.1)
Source				
	Urban	171 (59.8)	114 (55.6)	57 (70.4)
	Rural	115 (40.2)	91 (44.4)	24 (29.6)
Marital status				
	Cohabitant	258 (90.2)	190 (92.7)	68 (84.0)
	Married	24 (8.4)	12 (5.8)	12 (14.8)
	Separated/divorced	4 (1.4)	3 (1.5)	1 (1.2)
Educational level				
	Primary	19 (6.7)	16 (7.8)	3 (3.7)
	Secondary	166 (58.0)	134 (65.4)	32 (39.5)
	Superior	101 (35.3)	55 (26.8)	46 (56.8)
Employment status				
	Unemployment	197 (68.9)	150 (73.2)	47 (58.0)
	With employment	89 (31.1)	55 (26.8)	34 (42.0)
Economic situation				
	Low	191 (66.8)	145 (70.7)	46 (56.8)
	Medium	70 (24.5)	48 (23.4)	22 (27.2)
	High	25 (8.7)	12 (5.9)	13 (16.0)
Gestational trimester				
	First quarter	23 (8.0)	18 (8.8)	5 (6.2)
	II trimester	26 (9.1)	19 (9.3)	7 (8.6)
	III trimester	237 (82.9)	168 (81.9)	69 (85.2)
Number of gestations				
	Primigravida	97 (33.9)	58 (28.3)	39 (48.2)
	Multigravida	177 (61.9)	136 (66.3)	41 (50.6)
	Grand multigravida	12 (4.2)	11 (5.4)	1 (1.2)
Pregnancy planning				
	Yes	128 (44.8)	82 (40.0)	46 (56.8)
	No	158 (55.2)	123 (60.0)	35 (43.2)
Intimate partner violence				
	Yes	159 (55.6)	138 (67.3)	21 (25.9)
	No	127 (44.4)	67 (32.7)	60 (74.1)

### Gynecologic-obstetric Characteristics

Pregnant women from all trimesters of pregnancy participated, with a predominance of the third trimester (82.9%). Regarding the number of gestations, a higher proportion of multiparous women (61.9%) was observed. Additionally, 55.2% reported that their pregnancy was unplanned; this proportion was higher in the alexithymia group (60%), while in the non-alexithymia group, planned pregnancy predominated (56.8%).

55.6% of pregnant women reported having experienced IPV. This percentage was considerably higher among those with alexithymia (67.3%), in contrast to pregnant women without alexithymia, of whom 74.1% reported not having suffered violence (table 1).

### Bivariate analysis

The prevalence of alexithymia was found to be 65% higher in pregnant women who presented IPV compared to those who did not experience it (PR=1.65; 95% CI: 1.38-1.96; p<0.001), a statistically significant difference (table 2).

**Table 2:** Association of sociodemographic, gyneco-obstetric characteristics and intimate partner violence with alexithymia in pregnant women from two hospitals in Cusco.

Characteristics		Outcome: alexithymia		
		PRc	95% CI	p-value
Intimate partner violence				
	No	Ref.		
	Yes	1.65	1.38-1.96	<0.001
Age				
	18-34 years	Ref.		
	35 or more	1.12	0.94-1.34	0.190
Source				
	Urbano	Ref.		
	Rural	1.19	1.03-1.37	0.018
Marital Status				
	Married	Ref.		
	Cohabitant	1.47	0.98-2.21	0.062
	Separated/divorced	1.50	0.75-3.00	0.252
Educational attainment				
	Superior	Ref.		
	Primary	1.55	1.19-2.01	0.001
	Secondary	1.48	1.22-1.80	<0.001
Employment status				
	Employed	Ref.		
	Unemployment	1.23	1.03-1.48	0.024
Economic situation				
	High	Ref.		
	Medium	1.43	0.92-2.21	0.111
	Under	1.58	1.04-2.40	0.031
Gestational trimester				
	First quarter	Ref.		
	Second trimester	0.93	0.68-1.28	0.673
	III trimester	0.91	0.72-1.14	0.400
Number of pregnancies				
	Primigravida	Ref.		
	Multigravida	1,29	1.07-1.54	0.007
	Grand multigravida	1.53	1.21-1.94	<0.001
Pregnancy Planning				
	Yes	Ref.		
	No	1.22	1.04-1.42	0.013

PRc: crude prevalence ratio without adjustment for covariates; 95% CI: 95% confidence interval.

Regarding sociodemographic characteristics, pregnant women over 35 years of age showed a 12% higher prevalence of alexithymia compared to those in the 18 to 34 age group, although this difference was not statistically significant (PR=1.12; 95% CI: 0.94-1.34; p=0.190). Regarding geographical origin, a 19% higher prevalence of alexithymia was observed among pregnant women from rural areas compared to those from urban areas, a difference that was statistically significant (PR=1.19; 95% CI: 1.03-1.37; p=0.018) (table 2).

Regarding civil status, pregnant women with cohabiting, separated, or divorced status showed a prevalence of alexithymia 47% and 50% higher, respectively, compared to married women; however, these differences did not reach statistical significance.

Regarding educational level, pregnant women with primary education showed a 55% higher prevalence of alexithymia compared to those with higher education (PR=1.55; 95% CI: 1.19-2.01; p=0.001), a statistically significant difference. Similarly, pregnant women with secondary education presented a 48% higher prevalence compared to those with higher education (PR=1.48; 95% CI: 1.22-1.80; p<0.001), also a significant difference (Table 2).

Regarding the employment situation, unemployed pregnant women presented a 23% higher prevalence of alexithymia compared to employed women, a statistically significant difference (PR = 1.23; 95% CI: 1.03-1.48; p = 0.024).

In relation to the economic situation, pregnant women with low economic status presented a 58% higher prevalence, a statistically significant difference (PR = 1.58; 95% CI: 1.04-2.40; p = 0.031) (Table 2).

In the gynecological-obstetric field, the prevalence of alexithymia increased by 29% (PR=1.29; 95% CI: 1.07-1.54; p<0.007) in multigravidae and by 53% (PR=1.53; 95% CI: 1.21-1.94; p<0.001) in grand multigravidae, compared to primigravidae, these differences being statistically significant. Likewise, pregnant women who did not plan their pregnancy presented a 22% higher prevalence of alexithymia (PR=1.22; 95% CI: 1.04-1.42; p<0.013) compared to those who did plan it, a difference that was also statistically significant (table 2).

### Multivariate Analysis

Multivariate analysis showed that intimate partner violence increased the prevalence of alexithymia by 50% (adjusted PR: 1.50; 95% CI: 1.26-1.79; p<0.001) compared to those who did not suffer from intimate partner violence. This increase was statistically significant, after adjusting for sociodemographic and gynecological-obstetric variables, such as place of origin, educational level, employment status, economic situation, number of pregnancies, and pregnancy planning (table 3).

**Table 3:** Adjusted association of intimate partner violence with alexithymia in pregnant women from two hospitals in Cusco.

Characteristics		Outcome: alexithymia		
		Adjusted PR	CI 95%	p-value
	Intimate partner violence			
	No	Ref.		
	Yes	1.50	1.26 - 1.79	<0.001

PR: prevalence ratio; 95% CI: 95% confidence interval.

PR adjusted for place of origin, educational level, employment status, economic status, number of pregnancies, and pregnancy planning.

## DISCUSSION

In the present study, an elevated frequency of alexithymia and intimate partner violence was observed in the pregnant women evaluated. Likewise, a significant association was found between both variables, evidencing that pregnant women who reported intimate partner violence presented a 50% higher prevalence of alexithymia compared to those who did not experience violence, even after adjustment for sociodemographic and gynecological-obstetric variables. This result coincides with previous research that reports significantly higher levels of alexithymia in women victims of IPV ^11^^,^^16^^-^^20^

On the other hand, family violence is a prevalent public health problem in Cusco. The proportion of pregnant women who have experienced intimate partner violence (55%), exceeded the national average of 52.0% reported by ENDES 2024 in women of reproductive age ^4^, and is higher than that described in previous studies conducted in pregnant women, where the prevalence of violence ranged between 14% and 42% ^5^^,^^27^^,^^28^. These findings are consistent with recent national reports on violence against women, which continue to show a high burden of cases attended for violence during the year 2025 in the country ^29^. Likewise, the results of the Demographic and Family Health Survey (ENDES) 2024 confirm that violence against women continues to represent a frequent problem in the national context ^4^. However, these comparisons should be interpreted with caution due to methodological differences between population studies, administrative records, and specific hospital samples.

On the other hand, the frequency of alexithymia (71.7%) was considerably high, exceeding that reported by Linn *et al.* (40%) and Gilanifar *et al.* (50.8%) in pregnant women ^9^^,^^30^. Furthermore, it exceeds the values described in other populations, with values between 42% and 65% ^18^^,^^31^. This high frequency could be due to multiple factors, such as the emotional and psychological changes inherent to pregnancy ^32^, sociodemographic and psychosocial factors associated with acute or chronic stress situations ^33^^-^^35^.

While physiological and psychological changes are intrinsic to the adaptive process of gestation ^32^, in conditions of social, demographic, or emotional vulnerability, they can become risk factors that complicate pregnancy ^36^. In this context, the presence of alexithymia in pregnant women has been associated with a negative impact on the development of prenatal attachment ^21^ and a greater susceptibility to various psychopathological disorders, such as obsessive-compulsive symptoms, depression, anger, generalized anxiety, and phobic anxiety ^9^^,^^30^. Additionally, this condition could affect the neurobehavioral development of the fetus, increasing the risk of emotional and behavioral difficulties in childhood ^37^. These effects are potentiated when they coexist with situations of intimate partner violence, significantly compromising maternal health and that of the product ^6^^,^^7^^,^^15^.

Alexithymia can play a critical role in the cycle of violence ^20^^,^^34^. It has been described that exposure to traumatic experiences and chronic stress, frequent in contexts of violence, affects a person's ability to identify and express their emotions, inducing or exacerbating alexithymia ^35^. This, in turn, can act as a vulnerability factor by hindering effective communication and conflict resolution with the partner, thus contributing to a dysfunctional relational environment that favors the persistence of violence ^34^^,^^35^.

This dynamic is reinforced when considering gender differences, raised by previous studies that consider alexithymia to be more frequently associated with received violence than with exerted violence, a pattern more pronounced in women, who tend to present higher levels of alexithymia when they have been victims ^18^^,^^19^. In these cases, alexithymia could act as a risk factor for the emergence of reactive violence patterns ^18^. In contrast, in males, alexithymia is more related to exerted violence, possibly due to its negative influence on emotional regulation and increasing impulsivity, which predisposes to aggressive behaviors to exert violence ^38^. Therefore, alexithymia can play a dual role in the dynamic of intimate partner violence, by being a consequence and, at the same time, a factor that contributes to its perpetuation ^34^^,^^35^.

Mental health acquires special relevance in family care during pregnancy; however, it continues to be neglected due to various limitations in health systems ^39^, such as the lack of specialized resources and the limited integration of mental health into prenatal primary care ^40^^,^^41^.

In the Peruvian context, both mental health and domestic violence are considered national health priorities ^42^. The results of this study suggest that both problems can coexist during gestation, a stage of special vulnerability for women's physical and emotional well-being. The high frequency of intimate partner violence found and its association with alexithymia highlight the need to strengthen the comprehensive approach to mental health in prenatal care.

This need becomes especially relevant if it is considered that, in Peru, intimate partner violence has been related to adverse outcomes, including a higher probability of maternal and neonatal morbidity ^5^, which evidences that its effects transcend the psychological sphere and compromise health integrally. Likewise, recent studies suggest that alexithymia could play a relevant role in the dynamics of intimate partner violence, potentially favoring the persistence of violent relationships ^19^. Together, these findings support the need to strengthen the screening for intimate partner violence and emotional health indicators during prenatal check-ups, as well as the strengthening of articulated strategies among health, mental health, and violence protection services, especially in contexts where this problem maintains a high social and health burden.

It is important to point out some limitations of our study. Due to the cross-sectional design, it was not possible to establish causal relationships between IPV and alexithymia. Furthermore, the characteristics of the hospital population and the type of non-probabilistic sampling limit the generalization of the findings to other pregnant women. The measurement of violence could be subject to social desirability bias or underreporting, due to the sensitive nature of the topic; to minimize this, interviews were conducted ensuring privacy and confidentiality. Likewise, although the TAS-20 is widely used to assess alexithymia, previous studies in the Peruvian population have described limitations in its internal consistency and cultural equivalence ^43^, so the results should be interpreted with caution. Finally, the higher proportion of pregnant women in the third trimester and the absence of measurement of variables such as anxiety or depression could have influenced the observed association. Future studies should include the partner and evaluate relational and neurobiological factors involved in the perpetuation of the cycle of violence.

In conclusion, intimate partner violence is significantly associated with a higher prevalence of alexithymia in pregnant women. These findings suggest the importance of strengthening, during prenatal check-ups, timely violence detection strategies, emotional health assessment, articulation with mental health services, and protection against violence during prenatal care.
