# Supplementary material for: Intimate partner violence and its association with alexithymia in pregnant women from two hospitals in Cusco, Peru: an analytical cross-sectional study
Source: Rev Peru Med Exp Salud Publica. 2026 Jun 30;43(2):189–96. doi: 10.17843/rpmesp.2026.432.16064 (PMC13456924; doi:10.17843/rpmesp.2026.432.16064)
Supplement: Translation

# Translation: Violencia de pareja íntima y su asociación con alexitimia en gestantes de dos hospitales de Cusco, Perú: un estudio transversal analítico

**Authors:** Ana Claudia Santander-Cahuantico, Maycol Suker Ccorahua-Rios, Jorge Luis Cabezas Límaco, Victor Hugo Arias Chavez, Alejandro Joaquin Santander-Cahuantico, Franklin Miranda-Solis

**Affiliations:** 1 Escuela Profesional de Medicina Humana, Universidad Nacional de San Antonio Abad del Cusco, Cusco, Perú.; 2 Unidad de Cuidados Intermedios Adultos, Departamento de Emergencia y Cuidados Críticos, Hospital Antonio Lorena del Cusco, Cusco, Perú.

**Keywords:** Violencia de Pareja, Alexitimia, Salud Mental, Embarazadas

## Abstract

**Objetivos.:**

Determinar la asociación entre la violencia de pareja íntima y la alexitimia en gestantes atendidas en dos hospitales del Cusco, 2025.

**Materiales y métodos.:**

Se realizó un estudio transversal analítico, donde se incluyeron 286 gestantes atendidas en los consultorios externos de Ginecología y Obstetricia del Hospital Antonio Lorena y el Hospital Regional del Cusco entre febrero y abril del 2025. Se aplicaron entrevistas directas utilizando la Escala de Alexitimia de Toronto (TAS-20) y el Woman Abuse Screening Tool (WAST). El análisis estadístico se realizó mediante el uso del modelo lineal generalizado (GLM) con distribución de Poisson, función de enlace logarítmico y varianza robusta, calculando razones de prevalencia ajustadas (RPa).

**Resultados.:**

Se incluyeron 286 gestantes. El 71,7% presentó alexitimia y el 55,6% reportó violencia de pareja íntima (VPI). El estudio revela que la prevalencia de alexitimia fue 50% mayor en gestantes con VPI (RPa: 1,50; IC 95%: 1,26-1,79; p<0,001), ajustado por procedencia, grado de instrucción, situación laboral, situación económica, numero de gestaciones y la planificación del embarazo.

**Conclusiones.:**

La violencia de pareja intima se asoció significativamente con una mayor prevalencia de alexitimia en gestantes atendidas en dos hospitales del Cusco. Las gestantes expuestas a violencia presentaron una prevalencia 50% mayor de alexitimia, incluso después del ajuste por variables sociodemográficas y obstétricas.

## INTRODUCCIÓN

La violencia de pareja íntima (VPI) constituye un grave problema de salud pública debido a su alta prevalencia y a las múltiples consecuencias negativas que ocasiona en la salud física y mental de las víctimas ^1^. Según la Organización Panamericana de la Salud (OPS), aproximadamente el 25% de las mujeres de 15 a 49 años han experimentado VPI física y/o sexual al menos una vez en su vida ^2^. Esta carga es especialmente elevada en países de ingresos bajos y medianos, donde se concentra la mayor prevalencia de violencia contra la mujer ^3^. En el Perú, datos de la Encuesta Demográfica y de Salud Familiar (ENDES) 2024 reportaron que el 52,0% de las mujeres alguna vez sufrió algún tipo de VPI ^4^, lo que evidencia su importancia en el contexto nacional.

Esta problemática adquiere una dimensión aún más crítica durante el embarazo, ya que las gestantes constituyen una población particularmente vulnerable ^5^. Su exposición a la violencia se ha vinculado con complicaciones obstétricas, como trauma fetal, parto prematuro, aborto espontáneo, bajo peso al nacer y mayor riesgo de mortalidad materna; así como con problemas de salud mental, incluyendo un mayor riesgo de depresión, ansiedad, estrés postraumático y abuso de sustancias ^5^^-^^7^.

En este contexto, la alexitimia, entendida como la dificultad para identificar, describir y expresar emociones ^8^, emerge como un factor crucial. Ha sido descrita como un posible mecanismo de afrontamiento ante situaciones de estrés crónico, como la violencia, y podría mediar o exacerbar los efectos emocionales durante el embarazo ^9^. No obstante, aunque existe evidencia de una relación significativa entre VPI y alexitimia en mujeres adultas, la literatura aún no ha explorado esta asociación en mujeres gestantes ^10^^-^^12^.

Esta brecha en la investigación es particularmente relevante en el Perú, donde, pese a los esfuerzos institucionales por erradicar la VPI, los niveles siguen siendo preocupantes ^(^^4^. La situación es más crítica en regiones como Cusco, donde los niveles superan la media nacional y los factores económicos, sociales y culturales pueden amplificar los efectos de la violencia, así como limitar el acceso a servicios de salud mental adecuados ^13^. La escasez de estudios sobre la interrelación entre VPI y alexitimia durante la gestación en este contexto deja un vacío crítico de conocimiento. Esto constituye no solo un desafío constante para el sistema de salud nacional, sino también una preocupación global alineada con los Objetivos de Desarrollo Sostenible, que promueven la equidad de género y la erradicación de toda forma de violencia contra las mujeres ^14^. Comprender esta relación es urgente, dada la evidencia que sugiere un vínculo entre VPI y niveles elevados de alexitimia ^15^^-^^20^ y sus implicaciones negativas en la salud mental y en la calidad del vínculo materno-fetal ^21^.

Por lo tanto, este estudio tuvo como objetivo determinar la asociación entre la violencia de pareja íntima y la alexitimia en gestantes atendidas en dos hospitales del Cusco.

**MENSAJES CLAVE**

Motivación para realizar el estudio. Los altos índices de violencia de pareja durante el embarazo y la escasa evidencia sobre su impacto en la capacidad para procesar emociones (alexitimia) motivaron la realización de esta investigación.

Principales hallazgos. El estudio encontró una clara asociación entre sufrir violencia y presentar alexitimia en el embarazo, vinculando directamente el maltrato con dificultades emocionales.

Implicancias en salud pública. Estos hallazgos evidencian la necesidad de fortalecer el cumplimiento de las estrategias nacionales de detección y atención integral de la violencia contra la mujer durante el control prenatal.

## MATERIALES Y MÉTODOS

### Diseño y población de estudio

Se realizó un estudio observacional, trasversal analítico elaborado según las pautas de Strengthening the Reporting of Observational Studies in Epidemiology (STROBE^) (^^22^, en gestantes atendidas en los consultorios externos de Ginecología y Obstetricia de dos hospitales públicos de nivel III del Cusco pertenecientes al Ministerio de Salud (MINSA). Ambos hospitales fueron seleccionados por ser centros de referencia regional que concentran una alta demanda de atención con características asistenciales comparables. La recolección de información se realizó entre febrero y abril de 2025.

La población estuvo conformada por gestantes mayores de 18 años que otorgaron su consentimiento informado. Se excluyeron aquellas con deterioro cognitivo, complicaciones obstétricas graves, hospitalización o atención por emergencia. El tamaño muestral fue calculado mediante el programa Epi Info™ versión 7.2.5.0 para un estudio analítico transversal de comparación de proporciones. Debido a la ausencia de estudios locales previos que evaluaron la asociación entre violencia de pareja íntima y alexitimia en gestantes, se utilizó como referencia los parámetros reportados por Chandan *et al*. ^23^, considerando un nivel de confianza del 95%, un poder estadístico del 80%, una razón no expuestas/expuestas de 4:1, una proporción esperada del desenlace de 23,9% en no expuestas y 45,1% en expuestas. Se obtuvo un tamaño mínimo de 260 participantes, Adicionalmente, se incrementó un 10% por posibles pérdidas, obteniéndose finalmente una muestra de 286 gestantes. Las participantes fueron incluidas mediante muestreo no probabilístico consecutivo.

### Recolección de datos

La recolección de información se realizó entre febrero y abril de 2025 en los consultorios externos de Ginecología y Obstetricia de los hospitales participantes. Las gestantes fueron invitadas a participar al finalizar su consulta prenatal. Antes de su inclusión en el estudio, se verificó el cumplimiento de los criterios de elegibilidad y se obtuvo el consentimiento informado por escrito.

Durante el periodo de estudio se invitó a participar a 297 gestantes que cumplían los criterios de elegibilidad. Ocho gestantes declinaron participar luego de recibir la información sobre el estudio. De las 289 que aceptaron participar y completaron el proceso de consentimiento informado, tres fueron excluidas del análisis debido a información incompleta en los cuestionarios. Finalmente, se incluyeron 286 gestantes.

La aplicación de los instrumentos estuvo a cargo de encuestadores previamente capacitados en los objetivos del estudio, la administración estandarizada de los cuestionarios y el manejo de información sensible relacionada con violencia de pareja íntima. Las entrevistas se realizaron de forma individual en un ambiente independiente del área de consulta, garantizando privacidad y confidencialidad durante todo el proceso.

El tiempo promedio de aplicación fue de aproximadamente 15 minutos por participante. Al finalizar cada entrevista, los cuestionarios fueron revisados por los encuestadores para verificar la integridad de la información recolectada.

### Instrumentos y variables

La recolección de datos se efectuó a través de una encuesta distribuida en las siguientes secciones: características sociodemográficas y gineco-obstétricas, la Escala de Alexitimia de Toronto (TAS-20) y el Woman Abuse Screening Tool (WAST).

Las variables sociodemográficas incluyeron la edad de la gestante, lugar de procedencia, estado civil, grado de instrucción, situación laboral y situación económica. La procedencia fue clasificada como urbana o rural según el lugar de residencia habitual declarado por la gestante. La situación económica fue evaluada mediante autorreporte del ingreso mensual aproximado del hogar y categorizada en nivel bajo (1300-2400 soles), medio (2401-6999 soles) y alto (≥ 7000 soles). El trimestre gestacional se registró según la edad gestacional estimada por fecha de última menstruación o ecografía obstétrica. El número de gestaciones fue determinado mediante autorreporte de la participante e incluyó el embarazo actual. La planificación del embarazo por autorreporte, correspondió a la valoración de si el embarazo actual fue deseado y planificado antes de la concepción.

La variable de desenlace fue la Alexitimia, evaluada mediante la TAS-20 versión en español validada por Perez-Rincon *et al*. ^24^; compuesta por veinte ítems, y presenta un alfa de Cronbach de 0,875, lo cual indica alta consistencia interna. La puntuación total varia de 0 a 100, clasificándose en: ausencia de alexitimia (≤51 puntos), alexitimia limite (52‒60 puntos), y alexitimia (≥61 puntos) ^24^. Para el análisis estadístico, se dicotomizaron los resultados en dos categorías: ausencia de alexitimia (≤51 puntos) y presencia de alexitimia (≥52 puntos).

Para la evaluación de la VPI se aplicó el instrumento WAST (Woman Abuse Screening Tool) en su versión corta ^25^, adaptado al contexto peruano e incorporada por el MINSA en la Guía Técnica para el Cuidado de la Salud Mental de Mujeres en Situación de Violencia (2021) ^26^. Esta versión consta de ocho ítems con formato tipo Likert, con sensibilidad del 100%, especificidad del 96,4% y un alfa de Cronbach de 0,91 ^25^. El puntaje total varía entre 8 y 24 puntos. Se consideró tamizaje positivo para violencia de pareja íntima un puntaje ≥15. Adicionalmente, siguiendo los criterios de interpretación del instrumento, también se clasificó como positivo a las participantes que respondieron afirmativamente a las preguntas relacionadas con violencia física («Las discusiones ¿terminan en golpes, patadas o empujones?») o violencia verbal («Su pareja ¿la insulta, grita, humilla o descalifica verbalmente?»), incluso cuando el puntaje total era menor de 15 ^26^.

### Análisis estadístico

Los datos obtenidos fueron procesados en Microsoft Excel® 365 y analizados mediante el software Stata v15.1 (StataCorp, College Station, TX, USA). Se realizó un control de calidad para verificar la ausencia de datos faltantes («missing»), duplicados, celdas vacías o inconsistencias en la base de datos.

En el análisis univariado, se calcularon las frecuencias absolutas y relativas de las variables de estudio. Posteriormente, en el análisis bivariado, se evaluó la asociación entre violencia de pareja íntima y alexitimia, así como con las variables sociodemográficas y gineco-obstétricas. Para ello, se empleó un modelo lineal generalizado (GLM) con distribución de Poisson, función de enlace logarítmico y varianza robusta, a fin de estimar las razones de prevalencia (RP) y sus respectivos intervalos de confianza al 95%. Posteriormente, se realizó el análisis multivariado mediante el mismo modelo para obtener las RP ajustadas entre la violencia de pareja íntima y la alexitimia. Se incluyeron en el modelo ajustado las variables: procedencia, grado de instrucción, situación laboral, situación económica, numero de gestaciones y la planificación del embarazo. que mostraron asociación estadísticamente significativa en el análisis bivariado (p<0,05). Asimismo, se evaluó la multicolinealidad mediante el factor de inflación de la varianza (VIF), considerándose aceptables valores <6. Se consideró significativo un valor de p < 0,05.

### Aspectos éticos

El estudio contó con la autorización institucional del Hospital Regional del Cusco y del Hospital Antonio Lorena, fué evaluado y aprobado por el Comité Institucional de Ética en Investigación y la Oficina de Capacitación, Docencia e Investigación (código: 1107-HRC-OCDI). Asimismo, se obtuvo el consentimiento informado de todas las participantes, garantizando el anonimato y la confidencialidad de la información. A las gestantes que presentaron VPI y alexitimia se les brindó orientación sobre los servicios institucionales disponibles y se les recomendó seguimiento psicológico por parte del servicio de Psicología de los establecimientos participantes.

## RESULTADOS

Durante el periodo de reclutamiento se evaluaron 297 gestantes elegibles para participar en el estudio. Ocho rechazaron participar y tres fueron excluidas por presentar información incompleta en los cuestionarios. Finalmente, se incluyeron 286 gestantes atendidas en los servicios de consultorio externo de Ginecología y Obstetricia del Hospital Antonio Lorena (58,4%) y del Hospital Regional del Cusco (41,6%). Del total de participantes, el 71,7% (n = 205) presentó alexitimia, mientras que el 28,3% (n=81) no la presentó.

### Características sociodemográficas

El 85% de las participantes pertenecía al grupo etario de 18 a 34 años, proporción similar entre las gestantes con y sin alexitimia. Predominó la procedencia del área urbana (59,8%), sin diferencias marcadas entre ambos grupos. En cuanto al estado civil, la mayoría se identificó como conviviente (90,2%).

Respecto al grado de instrucción, el 58% de las gestantes había cursado educación secundaria, mientras que el 35,3% contaba con estudios superiores. Se observó que el 65,4% del grupo de gestantes con alexitimia tenía nivel secundario, en tanto que, entre las gestantes sin alexitimia, predominó la educación superior (56,8%). En relación con la situación laboral, la mayoría se encontraba desempleada (68,9%) y pertenecía a un estrato económico bajo (66,8%) (tabla 1).

**Tabla 1:** Distribución de las características sociodemográficas, gineco-obstétricas y de violencia de pareja íntima en gestantes, según presencia de alexitimia (N=286).

Características		Muestra total N=286	Gestante con alexitimia n=205	Gestante sin alexitimia n=81
		n (%)	n (%)	n (%)
Hospitales				
	H. Regional del Cusco	119 (41,6)	83 (40,5)	36 (44,4)
	H. Antonio Lorena	167 (58,4)	122 (59,5)	45 (55,6)
Edad				
	18-34 años	243 (85,0)	171 (83,4)	72 (88,9)
	35 a más	43 (15,0)	34 (16,6)	9 (11,1)
Procedencia				
	Urbano	171 (59,8)	114 (55,6)	57 (70,4)
	Rural	115 (40,2)	91 (44,4)	24 (29,6)
Estado civil				
	Conviviente	258 (90,2)	190 (92,7)	68 (84,0)
	Casada	24 (8,4)	12 (5,8)	12 (14,8)
	Separada/divorciada	4 (1,4)	3 (1,5)	1 (1,2)
Grado de instrucción				
	Primaria	19 (6,7)	16 (7,8)	3 (3,7)
	Secundaria	166 (58,0)	134 (65,4)	32 (39,5)
	Superior	101 (35,3)	55 (26,8)	46 (56,8)
Situación laboral				
	Sin empleo	197 (68,9)	150 (73,2)	47 (58,0)
	Con empleo	89 (31,1)	55 (26,8)	34 (42,0)
Situación económica				
	Bajo	191 (66,8)	145 (70,7)	46 (56,8)
	Medio	70 (24,5)	48 (23,4)	22 (27,2)
	Alto	25 (8,7)	12 (5,9)	13 (16,0)
Trimestre de gestación				
	I trimestre	23 (8,0)	18 (8,8)	5 (6,2)
	II trimestre	26 (9,1)	19 (9,3)	7 (8,6)
	III trimestre	237 (82,9)	168 (81,9)	69 (85,2)
Número de gestaciones				
	Primigesta	97 (33,9)	58 (28,3)	39 (48,2)
	Multigesta	177 (61,9)	136 (66,3)	41 (50,6)
	Gran multigesta	12 (4,2)	11 (5,4)	1 (1,2)
Planificación del embarazo				
	Sí	128 (44,8)	82 (40,0)	46 (56,8)
	No	158 (55,2)	123 (60,0)	35 (43,2)
Violencia de pareja íntima				
	Sí	159 (55,6)	138 (67,3)	21 (25,9)
	No	127 (44,4)	67 (32,7)	60 (74,1)

### Características gineco-obstétricas

Participaron gestantes de todos los trimestres del embarazo, con predominio del tercer trimestre (82,9%). En cuanto al número de gestaciones, se observó una mayor proporción de multigestas (61,9%). Además, el 55,2% refirió que su embarazo no fue planificado; esta proporción fue mayor en el grupo con alexitimia (60%), mientras que en el grupo sin alexitimia predominó el embarazo planificado (56,8%).

El 55,6% de las gestantes reportó haber experimentado VPI. Este porcentaje fue considerablemente mayor entre aquellas con alexitimia (67,3%), en contraste con las gestantes sin alexitimia, de las cuales el 74,1% refirió no haber sufrido violencia (tabla 1).

### Análisis bivariado

Se encontró que la prevalencia de alexitimia fue 65% mayor en las gestantes que presentaron VPI en comparación con aquellas que no la experimentaron (RP=1,65; IC95%: 1,38-1,96; p<0,001), diferencia estadísticamente significativa (tabla 2).

**Tabla 2:** Asociación de características sociodemográficas, gineco-obstétricas y violencia de pareja íntima con la alexitimia en gestantes de dos hospitales del Cusco.

Características		Desenlace: alexitimia		
		RPc	IC 95%	Valor de p
Violencia de pareja íntima				
	No	Ref.		
	Sí	1,65	1,38-1,96	<0,001
Edad				
	18-34 años	Ref.		
	35 a más	1,12	0,94-1,34	0,190
Procedencia				
	Urbano	Ref.		
	Rural	1,19	1,03-1,37	0,018
Estado Civil				
	Casada	Ref.		
	Conviviente	1,47	0,98-2,21	0,062
	Separada/divorciada	1,50	0,75-3,00	0,252
Grado de instrucción				
	Superior	Ref.		
	Primaria	1,55	1,19-2,01	0,001
	Secundaria	1,48	1,22-1,80	<0,001
Situación laboral				
	Con empleo	Ref.		
	Sin empleo	1,23	1,03-1,48	0,024
Situación económica				
	Alto	Ref.		
	Medio	1,43	0,92-2,21	0,111
	Bajo	1,58	1,04-2,40	0,031
Trimestre de gestación				
	I trimestre	Ref.		
	II trimestre	0,93	0,68-1,28	0,673
	III trimestre	0,91	0,72-1,14	0,400
Número de gestaciones				
	Primigesta	Ref.		
	Multigesta	1,29	1,07-1,54	0,007
	Gran multigesta	1,53	1,21-1,94	<0,001
Planificación del embarazo				
	Sí	Ref.		
	No	1,22	1,04-1,42	0,013

RPc: razón de prevalencia cruda sin ajuste por covariables; IC 95%: intervalo de confianza al 95%.

En cuanto a las características sociodemográficas, las gestantes mayores de 35 años presentaron una prevalencia de alexitimia 12% superior respecto a las del grupo de 18 a 34 años, aunque esta diferencia no fue estadísticamente significativa (RP=1,12; IC95%: 0,94-1,34; p=0,190). En relación con la procedencia geográfica, se observó una prevalencia 19% mayor de alexitimia entre las gestantes provenientes de zonas rurales en comparación con las de zona urbana, diferencia que resultó estadísticamente significativa (RP=1,19; IC95%: 1,03-1,37; p=0,018) (tabla 2).

Respecto al estado civil, las gestantes con condición de conviviente, separada o divorciada presentaron una prevalencia de alexitimia 47% y 50% mayor, respectivamente, en comparación con las casadas; sin embargo, estas diferencias no alcanzaron significancia estadística.

En cuanto al grado de instrucción, las gestantes con educación primaria mostraron una prevalencia de alexitimia 55% mayor frente a aquellas con educación superior (RP=1,55; IC95%: 1,19-2,01; p=0,001), diferencia estadísticamente significativa. De igual manera, las gestantes con educación secundaria presentaron una prevalencia 48% mayor en comparación con las de educación superior (RP=1,48; IC95%: 1,22-1,80; p<0,001), diferencia también significativa (tabla 2).

Respecto a la situación laboral, las gestantes sin empleo presentaron una prevalencia de alexitimia 23% mayor en comparación con las empleadas, diferencia estadísticamente significativa (RP = 1,23; IC95%: 1,03-1,48; p = 0,024).

En relación con la situación económica, las gestantes con nivel económico bajo presentaron una prevalencia 58% mayor, diferencia estadísticamente significativa (RP = 1,58; IC95%: 1,04-2,40; p = 0,031) (tabla 2).

En el ámbito gineco-obstétrico, la prevalencia de alexitimia aumentó un 29% (RP=1,29; IC95%: 1,07-1,54; p<0,007) en las multigestas y un 53% (RP=1,53; IC95%: 1,21-1,94; p<0,001) en las gran multigestas, en comparación con las primigestas, siendo estas diferencias estadísticamente significativas. Asimismo, las gestantes que no planificaron su embarazo presentaron una prevalencia de alexitimia un 22% mayor (RP=1,22; IC95%: 1,04-1,42; p<0,013) en comparación con aquellas que sí lo planificaron, diferencia que también resultó estadísticamente significativa (tabla 2).

### Análisis multivariado

El análisis multivariado mostró que la violencia por la pareja íntima aumentó la prevalencia de alexitimia en un 50% (RP ajustado: 1,50; IC 95%: 1,26-1,79; p<0,001) en comparación con aquellas que no padecían violencia por la pareja íntima. Este incremento resultó estadísticamente significativo, tras ajustar por variables sociodemográficas y gineco-obstétricas, como lugar de procedencia, grado de instrucción, situación laboral, situación económica, número de gestaciones y planificación del embarazo (tabla 3).

**Tabla 3:** Asociación ajustada violencia de pareja íntima con la alexitimia en gestantes de dos hospitales del Cusco.

Características		Desenlace: alexitimia		
		RP ajustado	IC 95%	Valor de p
	Violencia de pareja íntima			
	No	Ref.		
	Sí	1,50	1,26 - 1,79	<0,001

RP: razón de prevalencia; IC 95%: intervalo de confianza al 95%.

RP ajustado por lugar de procedencia, grado de instrucción, situación laboral, situación económica, número de gestaciones y planificación del embarazo.

## DISCUSIÓN

En el presente estudio se observó una elevada frecuencia de alexitimia y violencia de pareja íntima en las gestantes evaluadas. Asimismo, se encontró una asociación significativa entre ambas variables, evidenciándose que las gestantes que reportaron violencia de pareja íntima presentaron una prevalencia 50% mayor de alexitimia en comparación con aquellas que no experimentaron violencia, incluso después del ajuste por variables sociodemográficas y gineco-obstétricas. Este resultado coincide con investigaciones previas que reportan niveles significativamente más altos de alexitimia en mujeres víctimas de VPI ^11^^,^^16^^-^^20^

Por otra parte, la violencia familiar es un problema de prevalente de salud pública en el Cusco. La proporción de gestantes que ha experimentado violencia de pareja íntima (55,%), superó el promedio nacional del 52,0% reportado por la ENDES 2024 en mujeres en edad fértil ^4^, y es mayor a lo descrito en estudios previos realizado en gestantes, donde la prevalencia de violencia osciló entre el 14% y 42% ^5^^,^^27^^,^^28^. Estos hallazgos son consistentes con los reportes nacionales recientes sobre violencia contra la mujer, los cuales continúan evidenciando una elevada carga de casos atendidos por violencia durante el año 2025 en el país ^29^. Asimismo, los resultados de la Encuesta Demográfica y de Salud Familiar (ENDES) 2024 ratifican que la violencia contra la mujer continúa representando una problemática frecuente en el contexto nacional ^4^. No obstante, estas comparaciones deben interpretarse con cautela debido a las diferencias metodológicas entre estudios poblacionales, registros administrativos y muestras hospitalarias específicas.

Por otra parte, la frecuencia de alexitimia (71,7%) fue considerablemente alta, superando lo reportado por Linn *et al.* (40%) y Gilanifar *et al.* (50,8%) en gestantes ^9^^,^^30^. Además, supera los valores descritos en otras poblaciones, con valores entre el 42% y 65% ^18^^,^^31^. Esta elevada frecuencia podría deberse a múltiples factores, como los cambios emocionales y psicológicos propios de la gestación ^32^, factores sociodemográficos y psicosociales que están asociados a situaciones de estrés agudo o crónico ^33^^-^^35^.

Si bien los cambios fisiológicos y psicológicos son intrínsecos al proceso adaptativo de la gestación ^32^, en condiciones de vulnerabilidad social, demográfica o emocional, pueden convertirse en factores de riesgo que complican el embarazo ^36^. En este contexto, la presencia de alexitimia en mujeres gestantes se ha asociado con un impacto negativo en el desarrollo del apego prenatal ^21^ y una mayor susceptibilidad a diversos trastornos psicopatológicos, tales como síntomas obsesivo-compulsivos, depresión, ira, ansiedad generalizada y ansiedad fóbica ^9^^,^^30^. Además, esta condición podría afectar el desarrollo neuroconductual del feto, incrementando el riesgo de dificultades emocionales y conductuales en la infancia ^37^. Estos efectos se potencian cuando coexisten con situaciones de violencia de pareja, comprometiendo significativamente la salud materna y la del producto ^6^^,^^7^^,^^15^. 

La alexitimia puede desempeñar un papel critico en el ciclo de la violencia ^20^^,^^34^. Se ha descrito que la exposición a experiencias traumáticas y el estrés crónico, frecuentes en contextos de violencia, afecta la capacidad de una persona para identificar y expresar sus emociones, induciendo o exacerbando la alexitimia ^35^. Esta, a su vez, puede actuar como un factor de vulnerabilidad al dificultar la comunicación efectiva y la resolución de conflictos con la pareja, contribuyendo así a un entorno relacional disfuncional que favorece la persistencia de la violencia ^34^^,^^35^. 

Esta dinámica se refuerza al considerar las diferencias de género, planteada por estudios previos que consideran que la alexitimia se asocia con mayor frecuencia a la violencia recibida que a la ejercida, patrón más pronunciado en mujeres, quienes suelen presentar mayores niveles de alexitimia cuando han sido víctimas ^18^^,^^19^. En estos casos, la alexitimia podría actuar como factor de riesgo para la aparición de patrones de violencia reactiva ^18^. En contraste, en varones, la alexitimia se relaciona más con la violencia ejercida posiblemente debido a su influencia negativa en la regulación emocional y aumentar la impulsividad, lo que predispone a conductas agresivas para ejercer violencia ^38^. Por tanto, la alexitimia puede desempeñar un doble rol en la dinámica de la violencia de pareja, al ser una consecuencia y, a la vez, un factor que contribuye a su perpetuación ^34^^,^^35^.

La salud mental adquiere especial relevancia en el cuidado de la familia durante el embarazo; sin embargo, continúa siendo desatendida debido a diversas limitantes en los sistemas sanitarios ^39^, como la falta de recursos especializados y la limitada integración de la salud mental en la atención primaria prenatal ^40^^,^^41^.

En el contexto peruano, tanto la salud mental y violencia intrafamiliar son consideradas como prioridades nacionales de salud ^42^. Los resultados de este estudio sugieren que ambas problemáticas pueden coexistir durante la gestación, una etapa de especial vulnerabilidad para el bienestar físico y emocional de la mujer. La alta frecuencia de violencia de pareja íntima encontrada y su asociación con la alexitimia resaltan la necesidad de fortalecer el abordaje integral de la salud mental en la atención prenatal.

Esta necesidad cobra especial relevancia si se considera que, en el Perú, la violencia de pareja se ha relacionado con desenlaces adversos, incluyendo una mayor probabilidad de morbilidad materna y neonatal ^5^, lo que evidencia que sus efectos trascienden el ámbito psicológico y comprometen la salud de manera integral. Asimismo, estudios recientes sugieren que la alexitimia podría desempeñar un papel relevante en la dinámica de la violencia de pareja, favoreciendo potencialmente la persistencia de relaciones violentas ^19^. En conjunto, estos hallazgos respaldan la necesidad del fortalecer el tamizaje de violencia de pareja y de indicadores de salud emocional durante los controles prenatales, así como el fortalecimiento de estrategias articuladas entre los servicios de salud, salud mental y protección frente a la violencia, especialmente en contextos donde esta problemática mantiene una elevada carga social y sanitaria.

Es importante señalar algunas limitaciones de nuestro estudio. Debido al diseño transversal, no fue posible establecer relaciones de causalidad entre VPI y alexitimia. Además, las características de la población hospitalaria y el tipo de muestreo no probabilístico limitan la generalización de los hallazgos a otras gestantes. La medición de violencia podría estar sujeta a sesgo de deseabilidad social o subregistro, debido a la naturaleza sensible del tema; para minimizarlo, las entrevistas se realizaron garantizando privacidad y confidencialidad. Asimismo, aunque el TAS-20 es ampliamente utilizada para evaluar alexitimia, estudios previos en población peruana han descrito limitaciones en su consistencia interna y equivalencia cultural ^43^, por lo que los resultados deben interpretarse con cautela. Finalmente, la mayor proporción de gestantes en el tercer trimestre y la ausencia de medición de variables como ansiedad o depresión podrían haber influido en la asociación observada. Futuros estudios deberían incorporar a la pareja y evaluar factores relacionales y neurobiológicos involucrados en la perpetuación del ciclo de violencia.

En conclusion, la violencia de pareja íntima se asocia significativamente con una mayor prevalencia de alexitimia en gestantes. Estos hallazgos sugieren la importancia de fortalecer, durante los controles prenatales, las estrategias de detección oportuna de violencia y evaluación de la salud emocional y articulación con los servicios de salud mental, y protección frente a la violencia durante el control prenatal.
